# *slr2103*, a homolog of type-2 diacylglycerol acyltransferase genes, for plastoquinone-related neutral lipid synthesis and NaCl-stress acclimatization in a cyanobacterium, *Synechocystis* sp. PCC 6803

**DOI:** 10.3389/fpls.2023.1181180

**Published:** 2023-04-26

**Authors:** Mimari Kondo, Motohide Aoki, Kazuho Hirai, Taku Sagami, Ryo Ito, Mikio Tsuzuki, Norihiro Sato

**Affiliations:** School of Life Sciences, Tokyo University of Pharmacy and Life Sciences, Hachioji, Tokyo, Japan

**Keywords:** plastoquinone-related neutral lipid, saline stress, slr2103, Synechocystis, triacylglycerol, acyltransferase

## Abstract

A cyanobacterium, *Synechocystis* sp. PCC 6803, contains a lipid with triacylglycerol-like TLC mobility but its identity and physiological roles remain unknown. Here, on ESI-positive LC-MS^2^ analysis, it is shown that the triacylglycerol-like lipid (lipid X) is related to plastoquinone and can be grouped into two subclasses, X_a_ and X_b_, the latter of which is esterified by 16:0 and 18:0. This study further shows that a *Synechocystis* homolog of type-2 diacylglycerol acyltransferase genes, *slr2103*, is essential for lipid X synthesis: lipid X disappears in a *Synechocystis slr2103*-disruptant whereas it appears in an *slr2103*-overexpressing transformant (OE) of *Synechococcus elongatus* PCC 7942 that intrinsically lacks lipid X. The *slr2103* disruption causes *Synechocystis* cells to accumulate plastoquinone-C at an abnormally high level whereas *slr2103* overexpression in *Synechococcus* causes the cells to almost completely lose it. It is thus deduced that *slr2103* encodes a novel acyltransferase that esterifies 16:0 or 18:0 with plastoquinone-C for the synthesis of lipid X_b_. Characterization of the *slr2103*-disruptant in *Synechocystis* shows that *slr2103* contributes to sedimented-cell growth in a static culture, and to bloom-like structure formation and its expansion by promoting cell aggregation and floatation upon imposition of saline stress (0.3-0.6 M NaCl). These observations provide a basis for elucidation of the molecular mechanism of a novel cyanobacterial strategy to acclimatize to saline stress, and one for development of a system of seawater-utilization and economical harvesting of cyanobacterial cells with high-value added compounds, or blooming control of toxic cyanobacteria.

## Introduction

Photosynthetic microbes such as cyanobacteria and microalgae, which assimilate CO_2_ with the use of light energy, are promising bioresources for production of high-value added compounds for bioenergy, biodegradable plastics, health foods, and cosmetic production (Arora et al., 2021). Considerable interest has arisen regarding neutral lipids in photosynthetic microbes, i.e., algal triacylglycerol (TG) and cyanobacterial poly-β-hydroxybutyrate (PHB), which are raw materials for biodiesel and biodegradable plastics production, respectively. Carbon-neutral biodiesel derived from algae could help reduce global warming through replacement of fossil fuels that otherwise would lead to high CO_2_ emission levels and future fossil-fuel exhaustion. Meanwhile, production of biodegradable plastics would alleviate not only future exhaustion of fossil fuels but also microplastics pollution problems (e.g., Goncalves et al., 2016). Researchers have explored photosynthetic-microbe strains that have the potential to produce valuable compounds, and have genetically modified them to enhance their production ability (Hu et al., 2008; Goncalves et al., 2016). Besides establishment of strains and their growth conditions, economization of algal-cell culturing, harvesting, and biomass processing are prerequisites for industrialization of the production of valuable compounds (Min et al., 2022). It should be emphasized that the harvesting process, which generally comprises cell flocculation, dewatering, and drying, is particularly expensive owing to the high costs of energy and equipment (Min et al., 2022).

TG is physiologically crucial as an energy- and carbon-storage compound, and included in intracellular lipid droplets (LD) in eukaryotes in general and in prokaryotes of certain genera (Alvarez, 2016; Chapman et al., 2019). In eukaryotes, the terminal step of TG synthesis is catalyzed mainly by membrane-bound acyl-CoA:diacylglycerol (DG) acyltransferases (DGAT), which show two structurally distinct forms. One is DGAT1 in a superfamily of membrane-bound *O*-acyltransferases whereas the other is DGAT2 in a family that includes acyl-CoA:monoacylglycerol and acyl-CoA:wax alcohol acyltransferases (Liu et al., 2012). Moreover, a soluble DGAT3 has been found to contribute to TG synthesis in seed plants (Liu et al., 2012). Aside from DGAT1-3, phospholipids: diacylglycerol acyltransferase (PDAT) also transfers the acyl group at the *sn*-2 position of phospholipids to DG in yeasts and eukaryotic photosynthetic organisms, including plants and algae (Goncalves et al., 2016). Meanwhile, in bacterial strains that belong to the genera *Rhodococcus* and *Mycobacterium*, TG is synthesized by a bifunctional enzyme, i.e., wax ester synthase/acyl-CoA:diacylglycerol acyltransferase, which is structurally unrelated to either DGAT or PDAT (Daniel et al., 2004; Turkish and Sturley, 2009).

On TLC analysis, it was revealed that several cyanobacterial strains, including *Synechococcus elongatus* PCC 6301 (formally known as *Anacystis nidulans*, and almost equivalent to *Synechococcus elongatus* sp. PCC 7942), *Microcystis aeruginosa*, and *Oscillatoria rubescenes*, contained no TG and/or accumulated very little TG under nitrogen (N)-starved conditions, which is a well-known stressor to induce TG accumulation in algal species (e.g., Piorreck et al., 1984). In contrast, TG was reported to be found in some other cyanobacterial strains, including *Nostoc commune* and *Arthrospira platensis* (formally known as *Spirulina platensis*) (Taranto et al., 1993; Ramadan et al., 2008; Peramuna and Summers, 2014; Santana-Sánchez et al., 2021). However, TG was identified through TLC analysis only, with non-cyanobacterial phospholipids such as phosphatidylcholine and phophatidylethanolamine also being extracted in substantial amounts in some cases, leaving the possibility of contamination by some other organism(s) that intrinsically contain TG. In *Synechocystis* sp. PCC 6803 (herein referred to as *Synechocystis*), two research groups recently reported comigration of a lipid with marker TG on TLC analysis and the presence of TG on LC-MS analysis (Aizouq et al., 2020; Tanaka et al., 2020). Tanaka et al. (2020) further demonstrated that the TG content explained only 10% of the TG-like lipid found on TLC analysis, and did not greatly surpass the background level. In line, Tanaka et al. (2020) excluded the responsibility of *slr2103*, a homolog of DGAT2 in *Synechocystis*, for TG synthesis whereas Aizouq et al. (2020) reported that *slr2103*, which was found as a homolog of the phytyl ester synthase 2 (PES2) gene of *Arabidopsis thaliana* (Lippold et al., 2012), was responsible for the synthesis of both phytyl ester and TG. Independently of the above research on *Synechocystis*, we have long been aware of a neutral lipid in *Synechocystis* with similar TLC-mobility to that of TG during our research focused on algal TG synthesis (Shiratake et al., 2013; Sato et al., 2014; Hirai et al., 2016; Hayashi et al., 2017; Otaki et al., 2019; Oishi et al., 2022), and have investigated the chemical structure of the TG-like lipid and the function of *slr2103* in *Synechocystis*.

Here, distinct from the two groups that demonstrated TG in *Synechocystis*, it is concluded that TG is absent in *Synechocystis*; instead, the TG-like lipid represents a plastoquinone-related neutral lipid (designated here as lipid X), as revealed on LC-MS^2^. Moreover, we show the essentiality of *slr2103* for the synthesis of lipid X but not of TG, and identified Slr2103 as a novel acyltransferase that esterifies the hydroxy derivative of plastoquinone (plastoquinone-C; Kruk and Szymańska, 2021) with saturated fatty acids. In line, a novel acclimatization strategy as to NaCl stress in *Synechocystis* through formation of a bloom-like structure and essentiality of *slr2103* in the strategy became evident for *Synechocystis*. In this context, it should be mentioned that cyanobacterial blooms occur worldwide, which causes deterioration of aquatic ecosystems and water quality (Paerl and Otten, 2013). The results obtained allowed us to discuss the inclusion of lipid X but not TG in cyanobacteria and its physiological role in NaCl-stress tolerance, and the application of the bloom-like structure formation to technologies for the biomass-harvesting process and cyanobacterial-blooming control.

## Materials and methods

### Cyanobacterial strains, media, and growth conditions

The cyanobacterial strains used were *Synechocystis* sp. PCC 6803, *Synechococcus* sp. PCC 7942 (herein referred to as *Synechococcus*), and their mutants generated through insertion of a gene of interest by double crossover. The cells were cultured at 30°C in BG-11 with illumination (50 μmol photons m^−2^ s^−1^) in a glass tube with bubbling aeration, as described previously (Sato et al., 2000), or statically in a titer plate or test tubes. Cell growth was monitored by measuring the OD_730_ value or chlorophyll (Chl) content in the culture with a spectrophotometer DU 640 (Beckman).

### Preparation of lipids and analysis of their constituent fatty acids

As previously described (Sato et al., 2000), total lipids were extracted from cyanobacterial cells, according to the method of Bligh and Dyer (1959), and then separated as necessary into individual lipid classes by TLC. For isolation of lipid X from total lipids, TLC was performed with a solvent system of hexane/diethyl ether/acetate (70:30:1, by vol.), as in the case of algal TG (Otaki et al., 2019), which was followed by its extraction with a solvent system of chloroform/methanol (2:1). Two-dimensional TLC was performed for separation of total lipids into individual polar lipid classes (Sato et al., 2017b): the first development with a solvent system of chloroform/methanol/H_2_O (65:25:4 by vol.), and the second one with another solvent system of chloroform/methanol/conc. NH_3_ solution (13:7:1 by vol.). Total lipids or TLC-separated lipid classes were used for capillary GLC analysis to quantify their constituent fatty acids (Sato et al., 2017b) or for LC-MS and LC-MS^2^ analysis (see below). As a simple method to separate total lipids into lipid X and some polar lipid classes, two-step TLC was used: first, a solvent system of ChCl_3_/CH_3_OH/H_2_O (65:25:4) was used until the front line of the solvent reached half way up the TLC plate. Second, another solvent system of hexane/diethyl ether/acetate (70:30:1, by vol.) was used for a whole run TLC.

### Lipid profiling with a liquid chromatography-electrospray ionization-tandem mass spectrometry system

An LC-MS system composed of LC-20A Prominence series HPLC (Shimadzu, Kyoto, Japan), and a 3200 QTRAP hybrid triple quadrupole/linear ion trap mass spectrometer with a Turbo V™ ion source (Sciex, Concord, Ontario, Canada) was used for the lipid profiling of samples. Each sample (2 μL) was injected into an L-column 2 ODS (3 µm, 2.1 mm i.d. × 100 mm, CERI, Saitama, Japan) for reversed-phase separation of lipids into individual lipid classes and their molecular species by linear gradient elution as follows: mobile phases A (0.1% formic acid and 0.028% ammonium in 2-propanol/methanol/water at 2:2:1 (v/v/v)) and B (0.1% formic acid and 0.028% ammonium in 2-propanol), 30% B to 80% B in the first 25 min at a flow rate of 300 µL/min, followed by 80% B in the next 10 min. The column oven temperature was kept constant at 40°C. The ion source was operated in the positive ion mode with the following instrument parameters: curtain gas, 20 psi; ion spray voltage, 5,500 V; declustering potential, 40 V; temperature, 500°C; nebulizer gas, 60 psi; heater gas, 50 psi. The enhanced mass scan (EMS) mode was used for lipid signal survey. Tandem MS (MS^2^) analysis of lipid X molecular species was performed in the EMS mode with information dependent acquisition criteria, followed by enhanced product ion (EMS-IDA-EPI) scaning. The collision gas (CAD) value was set high with a collision energy (CE) value of 35 V. The data obtained were analyzed and quantified using Analyst 1.5.2 software (Sciex).

### LC-Q/TOF MS-based accurate-mass analysis

LC-Q/TOF MS analysis was carried out with the use of a 1290 infinity II high-performance liquid chromatograph and a 6530 Accurate-Mass Q-TOF mass spectrometer equipped with a JetStream source (both Agilent Technologies, Santa Clara, CA) for the identification of compounds. Chromatographic separation of the samples was performed under the same lipid profiling conditions to those above. The source was operated in the positive ionization mode as follows: ion spray 4000 V; gas temperature and sheath gas temperature 300 and 350°C, respectively; nebulizer (N2) 40 psi; and sheath gas flow 12 L/min. Data-dependent acquisition was used in the mass range of 50−1700 m/z for both MS and MS/MS with a collision energy of 25 V. The calibration of the spectral range was performed using Tuning & Performance Standards for the LC/MS solution (Agilent Technologies) and a fifth-order non-linear calibration curve was adopted. To perform the real-time lock mass correction, a lock mass solution including purine (m/z 121.0509) and HP-921 [hexakis (1H,1H,3H-tetrafluoro-pentoxy) phosphazene] (m/z 922.0098) was delivered by a built-in pump. The accurate mass Q-TOF MS and MS/MS data were processed with the use of MassHunter Workstation Software (Agilent Technologies).

### Gene manipulation in cyanobacteria

In *Synechocystis*, an ORF, *slr2103*, on the genome was disrupted, as previously described (Sato et al., 2000). A genomic region spanning the coding region of *slr2103* was amplified by PCR with primer set 1 (forward, 5’ AATACCATTCGCTCTAGCTG 3’; reverse, 5’ AATCCGGCCGTAGAACTGAC 3’). The PCR product was ligated to the pGEM T-EASY vector (Promega), cut with *Xho*I at the center, and then blunt-ended. The linear DNA obtained was ligated with the kanamycin-resistant gene for generation of a plasmid to disrupt *slr2103*, which was then used to transform wild-type (WT) cells of *Synechocystis*. The disruption was confirmed by genomic DNA PCR with primer set 1. Meanwhile, *slr2103* was overexpressed in *Synechococcus* with an expression vector, pTY1102_vktA, under the control of the ConII promoter of *Escherichia coli*, as previously described (Sato et al., 2017a). In brief, the coding region of *slr2103* was amplified with COD-plus DNA polymerase with primer set 2 (forward, 5’ GTGCTAAGAGCGACCAGTGA 3’; reverse, 5’ ATTCCAACAAACATCAGCGG 3’), and then ligated to the expression vector cut with *Sma*I in the correct orientation. The resultant plasmid and pTY1102_vktA were used for transformation of *Synechococcus* WT cells to obtain an *slr2103*-overexpressing transformant and its control, respectively. The transcript level of *slr2103* and that of *rnpB* as an internal control were investigated by semi-quantitative PCR with the use of primer sets 2, 3 (forward, 5’ AGTTAGGGAGGGAGTTGC 3’; reverse, 5’ TAAGCCGGGTTCTGTTCC 3’ for *Synechocystis*) and 4 (forward, 5’ GAAAGTCCGGGCTCCCAA 3’; reverse, 5’ TAAGCCGGGTTCTGTTCT 3’ for *Synechococcus*), respectively, as Sato et al. (2017a) described previously.

### Molecular phylogenetic tree

For phylogenetic analysis, the amino acid sequences of DGAT2 homologs were searched in available databases, including Cyanobase (https://genome.microbedb.jp/cyanobase/), with authentic DGAT2 sequences as queries. The sequences of DGAT2 homologs obtained are summarized in **Table S3**. The sequences were aligned after editing, including the deletion of regions with low conservation among these sequences, with ClustalX2 (Larkin et al., 2007) and SeaView (Gouy et al., 2010). The aligned sequences were subjected to phylogenetic analysis with ClustalX2 by the maximum-likelihood method (1000 bootstrap replicates).

## Results

### A novel neutral lipid in *Synechocystis*

**Figure 1:**
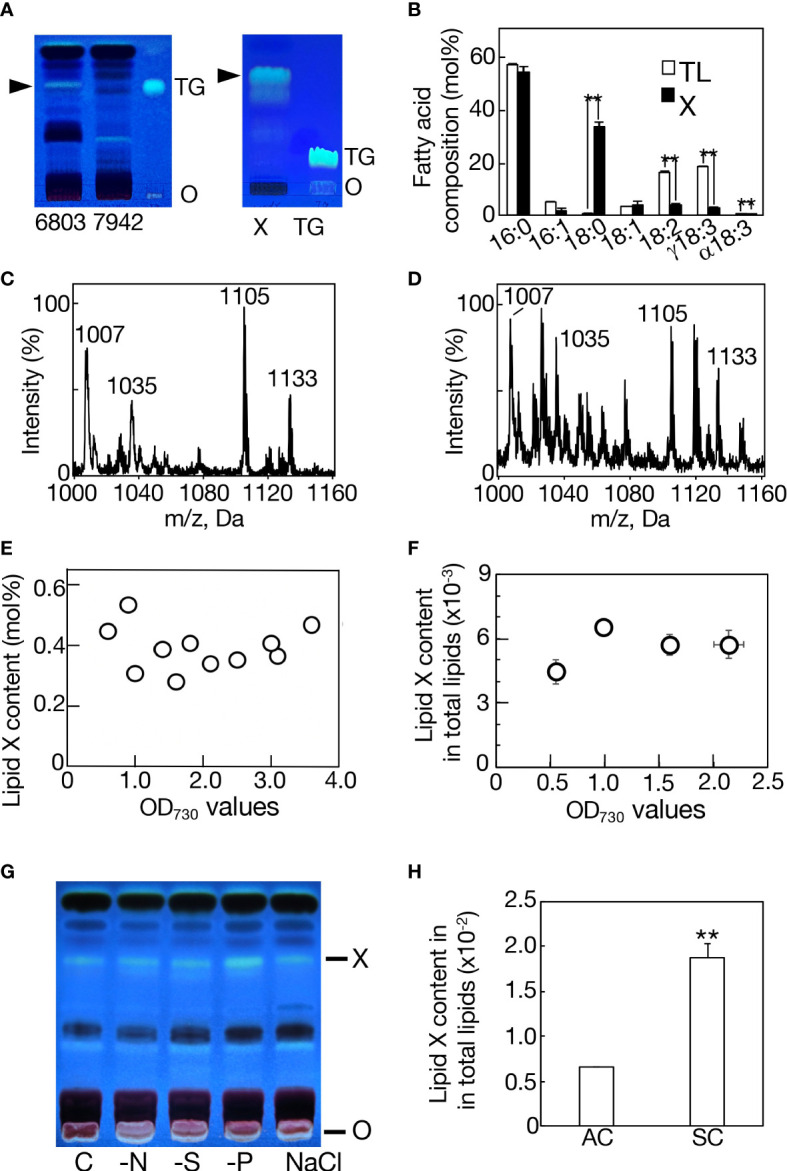
Characterization of lipid X in *Synechocystis*. **(A)** TLC profiles of total lipids in *Synechocystis* (6803) and *Synechococcus* (7942) with a solvent system of hexane/diethyl ether/acetate (70:30:1 by vol.) (left panel), and that of lipid X prepared from *Synechocystis* by TLC, as in the left panel, with another solvent system of 100% toluene (right panel). Arrowheads indicate the position of lipid X. O, origin. TG indicates a marker TG. **(B)** Fatty acid compositions of total lipids (TL) and lipid X (X). MS spectrum of TLC-prepared lipid X **(C)** or that of a lipid fraction (retention time of 15-17 min, m/z 1000-1160), including lipid X, in total lipids **(D)** on LC-MS analysis in *Synechocystis*. **(E)** Quantitative behavior of lipid X based on constituent fatty acid contents of TLC-prepared lipid X in total lipids on GC-analysis. **(F)** Quantitative behavior of a lipid fraction (retention time of 15-17 min, m/z 1000-1160), including lipid X, on LC-MS analysis of total lipids relative to total lipids (retention time of 2-18 min, m/z 200-1200) during cell growth in an aeration culture, based on the signal intensities on LC-MS analysis. **(G)** TLC profiles of lipid X in total lipids from aeration-cultured cells subjected to nitrogen-, sulfur- or phosphorus-deficiency (-N, -S or -P) stress, or 0.6 M NaCl stress. **(H)** Quantitative behavior of a lipid X fraction relative to total lipids in a static culture (SC) or in an aeration culture (AC), based on the signal intensities on LC-MS analysis, as in **(F)**. The values shown are averages ± SD for three experiments. The significance of differences was evaluated by means of Student’s *t*-test. **P<0.05.

Total lipids were isolated from bubbling-aeration cultured *Synechocystis* and *Synechococcus* cells, respectively, at the late logarithmic growth phase, and used for analysis of neutral lipids by TLC. On TLC analysis with the solvent system of hexane/diethyl ether/acetate (70:30:1 by vol.), a neutral lipid spot was observed with similar mobility to that of a marker TG in *Synechocystis* but not in *Synechococcus* (**Figure 1A**, left). The absence of TG in *Synechococcus* was compatible with a previous report (Piorreck et al., 1984). The neutral lipid of *Synechocystis* was extracted from the TLC plate, and then subjected to another TLC with a distinct solvent system of 100% toluene (**Figure 1A**, right). It turned out that this neutral lipid is not TG but a novel lipid, which is designated here as lipid X. The TLC-purified lipid X contained 16:0 and 18:0 as major fatty acids: the 16:0 content of lipid X was similar to that of total cellular lipids while the 18:0 content of lipid X was much higher than that of total lipids at the expense of unsaturated C_18_ fatty acids (**Figure 1B**). Meanwhile, the MS spectrum of the TLC-purified lipid X revealed four major signals of m/z 1007, 1035, 1105, and 1133 with several minor ones on positive-ESI LC-MS analysis (**Figure 1C**). In LC-MS analysis of total lipids, the MS spectrum of a lipid fraction (retention time of 15-17 min, m/z 1000-1160), including the above lipid X ions, revealed similar ion components to those of the TLC-purified lipid X, however, with distinct ratios of their signal intensities (**Figure 1D**). GC analysis of TLC-purified lipid X showed that the lipid X content stayed almost the same, occupying only 0.2-0.6 mol% of total lipids on a fatty acid basis, throughout the aeration culturing period, and even upon entry into the stationary growth phase (OD_730_ value > 2), where cells should encounter a variety of environmental stresses like nutritional depletion (**Figure 1E**). Similarly, it was demonstrated on LC-MS analysis of total lipids that lipid X in total was never markedly elevated during cell growth (**Figure 1F**). Consistently, drastic accumulation of lipid X was never observed in *Synechocystis* cells when subjected to N-, S- and P-starvation stresses, which are well known stressors that induce PHB accumulation (**Figure 1G**, Hirai et al., 2019). These results implied that lipid X, distinct from PHB, does not play a crucial role as a storage lipid that constructs lipid droplets. For simplification of the lipid sample preparation protocol and avoidance of possible denaturation of lipid X during the TLC-based purification process (DeLong et al., 2001), MS analysis of lipid X was subsequently performed with the use of total lipids.

### Classification of lipid X into two subclasses

For LC-MS analysis of lipid X, total lipids prepared from statically cultured cells in a microtiter plate were used owing to the more intense lipid X ion signals, relative to those from bubbling-aeration cultured cells (**Figure 1H**). The role of lipid X as a storage lipid should be excluded in this static culture also, since only less than a 3-fold increase was achieved in the content of lipid X (**Figure 1H**). Judging from the retention times in the LC-MS spectrum, the signals of lipid X could be divided into four groups, I-IV (**Supplemental Figure 1A**). Group I: m/z 1007, 1012, 1049, and 1105 at 15.2 min; group II: m/z 1021, 1026, 1063, and 1119 at 15.6 min; group III: m/z 1035, 1040, 1077, and 1133 at 15.9 min; and group IV: m/z 1049, 1054, 1091, and 1147 at 16.4 min.

**Figure 2:**
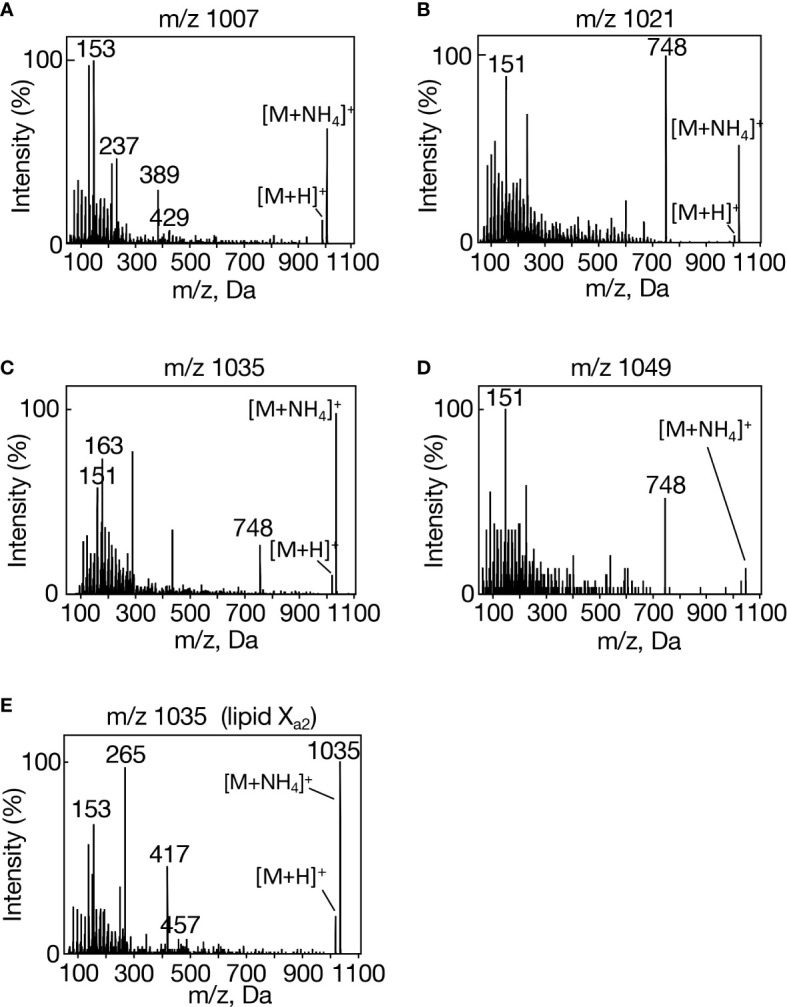
MS^2^ spectra of individual lipid X molecular species in *Synechocystis*. **(A)** m/z 1007, **(B)** m/z 1021, **(C)** m/z 1035, and **(D)** m/z 1049 in aeration-cultured cells, and **(E)** m/z 1035 in statically cultured cells.

In group I, m/z 1007 [M+NH_4_]^+^ showed a set of spiked signals below m/z 300 on MS^2^ analysis (**Figure 2A**). In group II, m/z 1021 [M+NH_4_]^+^exhibited similar spiked-signal ions to those in m/z 1007 and a unique m/z 748 ion (**Figure 2B**). Intriguingly, the MS^2^ spectra of both m/z 1035 [M+NH_4_]^+^ in group III and m/z 1049 [M+NH_4_]^+^ in group IV showed a similar set of fragment ions to those of m/z 1021, i.e., both spiked-signal ions and an m/z 748 one (**Figures 2C, D**). In view of inclusion of 16:0 and 18:0 in lipid X (**Figure 1B**), it was highly probable that m/z 748 in m/z 1021 and 1049 were due to neutral losses of 16:0 ([MH-C_15_H_31_COOH]^+^) and 18:0 ([MH-C_17_H_35_COOH]^+^), respectively. It however seemed least probable that m/z 748 in m/z 1035 resulted from a loss of 17:0 in view of the absence of 17:0 in lipid X (**Figure 1B**). Future study will be necessary to determine what was released from m/z 1035 to generate m/z 748. Of further note was the close resemblance in the fragment ion composition in the spiked signal region for m/z 1007, 1021, 1035, and 1049 (**Supplemental Table 1**). It would be reasonable to consider that in m/z 1021, 1035, and 1049, these spiked-signal fragment ions were derived from the lipid X structural part corresponding to fragment ion m/z 748, and therefore that the m/z 748 ions are the same in these three lipid X species. Similar to lipid X, carotenoids previously showed spiked-signal patterns in their MS^2^ spectra (van Breemen et al., 2012). Collectively, m/z 1007, 1021, 1035, and 1049 had some similar isoprenoid structure, and they could be divided into two subclasses, based on the presence or absence of m/z 748 as the fragment ion in their MS^2^ spectra. We thus designated m/z 1007, and m/z 1021, 1035, and 1049 as lipid X_a1_ and X_b1-3_, respectively.

It was possible that in individual groups I-IV, four ion members eluted at the same retention times on LC-MS represented four different ion-adducts of a lipid X molecular species. Moreover, it was of note that in group I, m/z 1012, 1049, and 1105 were greater than m/z 1007 [M+NH_4_]^+^ by m/z 5, 42, and 98, in that order, and that in individual groups II-IV, the same differences in m/z value were observed for their four ion members (**Supplemental Figure 1A**). In this context, it was highly likely that the m/z 1012 ion in group I corresponds to lipid X_a1_ [M+Na]^+^, compatible with extremely repressed fragmentation in its MS^2^ spectrum (**Supplemental Figure 2A**), and, similarly, m/z 1026, 1040, and 1054 would also represent [M+Na]^+^ in lipids X_b1_, X_b2_, and X_b3_, respectively (**Supplemental Figures 2B-D**). It should be emphasized that the m/z 1026, 1040, and 1054 ions showed a sole prominent fragment ion at m/z 770 (possibly, related to m/z 748 in m/z 1021, 1035, and 1049), distinct from m/z 1012, which exhibited almost no marked fragment ions: these results again strengthened our notion that lipid X can be divided into two subclasses, lipids X_a_ and X_b_.

Concerning m/z 1049, 1063, 1077, 1091, i.e., lipid X ion species that were greater than the corresponding [M+NH_4_]^+^ by m/z 42 in the individual groups, the MS^2^ spectra would also agree with the division of lipid X into two subclasses. m/z 1049 in group I, representing the lipid X_a_ subclass, showed a sole intense fragment ion, m/z 60, and m/z 1063, 1077, and 1091 in groups II-IV, representing the lipid X_b_ subclass, produced characteristic fragment ions, m/z 60, 748, and/or 807, together with the spiked-signal ions (**Supplemental Figure 2**). Meanwhile, only a single prominent fragment ion, m/z 116, was observed for m/z 1105, 1119, 1133, and 1147, respectively (**Supplemental Figure 2**). Collectively, with the use of total lipids, but not of TLC-prepared lipid X, to avoid artificial denaturation of lipid samples, it was successfully revealed that lipid X can be divided into two subclasses, lipids X_a_ and X_b_, and that lipid X_b_ comprises three molecular species, X_b1-3_.

### A homologous gene of *DGAT2* in *Synechocystis*

In an early stage of our investigation, the finding of the neutral lipid that showed TG-like mobility on TLC analysis (**Figure 1A**, left) prompted us to search for candidate genes for TG synthesis in its genome: homologous genes of known *DGAT1-3*, *PDAT* or wax ester synthase/acyl-CoA:diacylglycerol acyltransferase were searched for in its genome with the use of BLAST. As a result, *slr2103* was found as a sole candidate responsible for the final reaction step of TG synthesis, and was presumed to encode a protein that is 27, 27, 21, and 28% homologous to DGAT2 of a fungus, an animal, a seed plant, and a green alga, respectively (Cases et al., 2001; Lardizabal et al., 2001; Shockey et al., 2006; Hung et al., 2013; **Supplemental Figure 3**). The amino acid sequence of Slr2103, when aligned with those of known DGAT2s, indicated the six previously reported DGAT2 motifs (**Supplemental Figure 3A**). Motifs 1, 3, 4, and 5 were partially or well conserved in Slr2103: as to motif 1 (PH block), its core tetrapeptide, (H/E)PH(G/S), was converted into HNGG in Slr2103, whereas a core pentapeptide, GG(A/V)XE, in motif 3 (GGE block), was changed into GGAGD. Motif 4 (RGFA block) contains RXGF(V/I)(K/R)XA as a core peptide, which was completely conserved in Slr2103. In motif 5 (VPFG block), a core heptapeptide, VPXXXFG, was converted into VPAIAVG in *Slr2103*. Meanwhile, motifs 2 and 6 were less conserved in Slr2103: the regions corresponding to motif 2 (R block) and motif 6 (G block) did not contain a conserved residue, arginine or glycine, respectively (Cao, 2011). In addition to the six motifs, most DGAT2 sequences had a tripeptide, YFP, in the N-terminal region (Cao, 2011; Liu et al., 2012), which was substituted by YFR in Slr2103.

**Figure 3:**
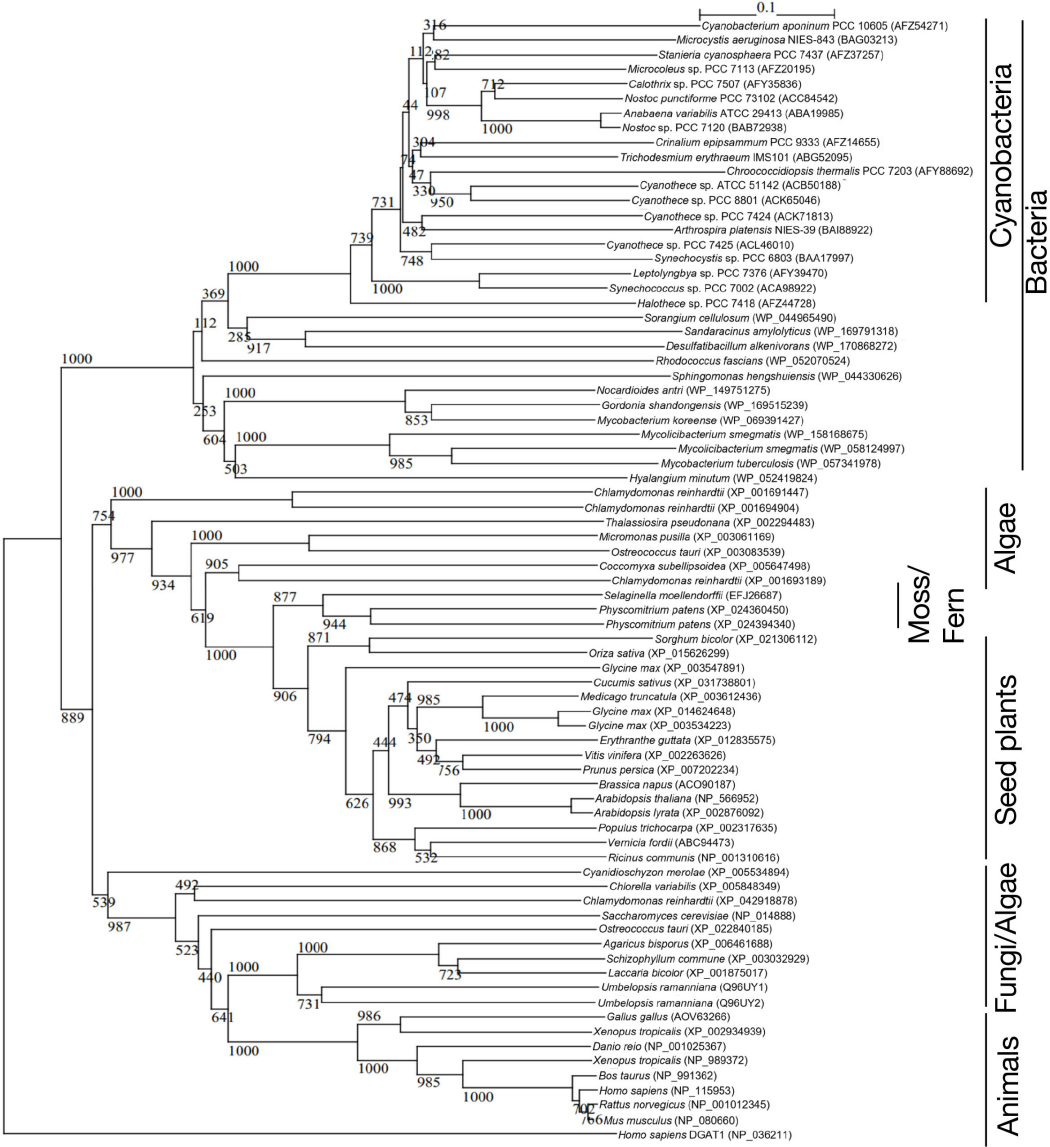
Phylogenetic tree of DGAT2 homologs. The tree was constructed with the use of sequence alignment of the DGAT2-homolog sequences shown in **Table S3**. Numbers at the branches: Bayesian posterior probabilities. The numbers after taxa: accession numbers.

DGAT2 is generally regarded as a membrane protein with one or two adjacent transmembrane domains at its N-terminus (Cao, 2011; Liu et al., 2012). According to SOSUI, a protein topological prediction program, however, it was predicted that Slr2103 has no membrane-spanning region and thus is a soluble protein (Imai et al., 2008; **Supplemental Figure 3B**). The same results were obtained for Slr2103 with another topological prediction program, TMHMM, based on a distinct algorithm (Krogh et al., 2001; **Supplemental Figure 3C**). A BLAST search in Cyanobase identified *slr2103* homologs in 103 cyanobacterial strains other than *Synechocystis*, with no homologs found in the other 265 strains (**Supplemental Table 2**). Notedly, molecular phylogenetic analysis indicated that Slr2103 and its cyanobacterial homologs constituted a clade, which was close to another clade that comprised bacteria sequences of unknown function, but was far from authentic DGAT2 sequences of eukaryotes, including photosynthetic organisms (**Figure 3**; **Supplemental Table 3**).

### Functional characterization of *slr2103*

**Figure 4:**
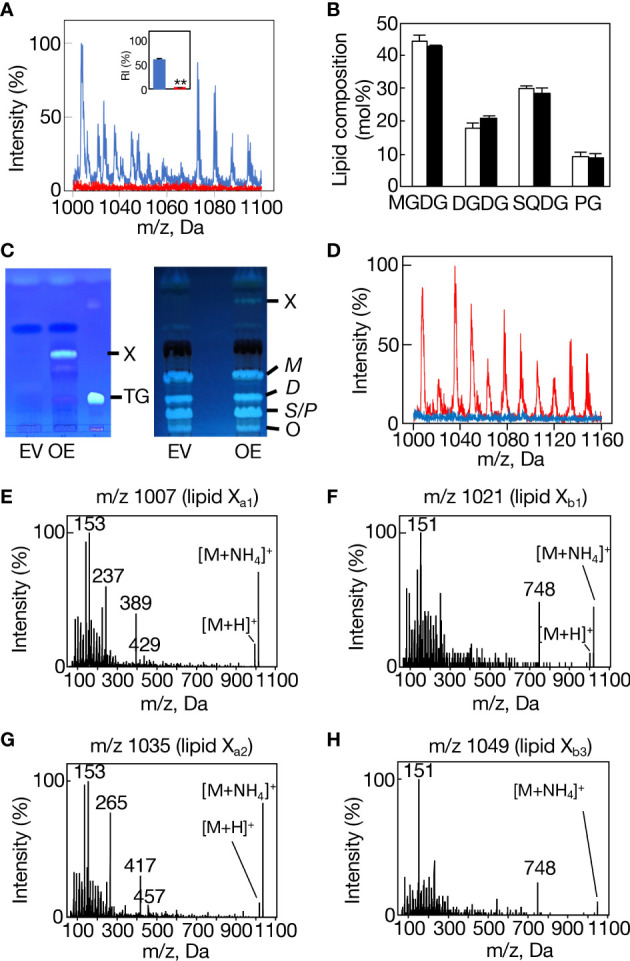
Loss-of-function and gain-of-function analyses of *slr2103*. **(A)** MS spectrum of a lipid fraction (retention time of 15-17 min, m/z 1000-1160), including lipid X, on LC-MS analysis of total lipids prepared from WT cells (blue) or *Δslr2103* ones (red). The signal intensity of m/z 1007 relative to a total lipid fraction (retention time of 2-18 min, m/z 200-1200) in the WT was adjusted to 100%. Inset, the estimated relative signal intensity of the lipid X fraction. The WT value was adjusted to 100%. **(B)** Polar lipid composition of *Δslr2103*. White and black bars indicate the WT and *Δslr2103*, respectively. **(C)** TLC-profiles of a lipid X fraction with a solvent system of 100% toluene (left). The lipid X fraction in OE or its counterpart in EV, which was obtained through TLC of total lipids with a solvent system of hexane/diethyl ether/acetate (70:30:1 by vol.), was then subjected to another TLC with a solvent system of 100% toluene. Note that OE but not EV showed lipid X that was quite distinct from a marker TG in mobility. The two-step TLC-profiles of total lipids (right). Note that the two-step TLC enabled not only separation of lipid X from polar lipids but also that of polar lipids into three fractions, MGDG (*M*), DGDG (*D*), and SQDG (*S*)+PG (*P*). **(D)** MS spectrum of the lipid fraction, including lipid X, on LC-MS analysis of total lipids prepared from EV cells (blue) or OE ones (red), with the signal intensity of m/z 1007 in OE relative to the total lipid fraction adjusted to 100%. MS^2^ spectrum of m/z 1007 **(E)**, m/z 1021 **(F)**, m/z 1035 **(G)**, or m/z 1049 **(H)**.

A disruptant as to *slr2103* (Δ*slr2103*) was generated in *Synechocystis* through insertion of a kanamycin-resistant gene cassette (**Supplemental Figure 4A**, see supplemental experimental procedures). Consistent with the absence of lipid X in Δ*slr2103* cells on TLC analysis of total lipids (**Supplemental Figure 4B**), we found the lack of a complete set of lipid X molecular species in Δ*slr2103* on LC-MS analysis of total lipids prepared from the aeration-cultured cells (**Figure 4A**). In contrast to lipid X, polar lipids were little affected in quantity in Δ*slr2103* relative to in the WT: MGDG was the most abundant, followed by SQDG, DGDG, and PG, in that order (**Figure 4B**). Slr2103 protein was thus dispensable for acylation in the synthesis of these polar lipids.

We then generated an *slr2103*-overexpressing transformant of *Synechococcus* (OE, **Figure 4C**, see supplemental experimental procedures), which, distinct from a control transformed with an empty vector (EV), was able to synthesize lipid X (**Figures 4C, D**). It should be emphasized that the overexpression of *slr2103* led to appearance of lipid X only but not of TG (**Figure 4C**, left). On LC-MS analysis of total lipids prepared from aeration-cultured cells, it seemed that lipid X in *Synechococcus* OE comprised the same four groups, I-IV, as those in *Synechocystis* WT, and that the respective groups contained the same four lipid X ion members as those in *Synechocystis* WT (**Supplemental Figures 1B, C, F**., **Supplemental Figure 1A**). Groups I, II, and IV were then confirmed to represent lipids X_a1_, X_b1_, and X_b3_ in MS^2^ spectra (**Figures 4E, F, H**, **Supplemental Table 1**, **Supplemental Figure 5**); e.g., observed were spiked-signal fragment ions in m/z 1007, 1021, and 1049, together with the fragment ion, m/z 748, of the prominent signal in only m/z 1021 and 1049, which was consistent with the idea that m/z 1007, 1021, and 1049 correspond to lipids X_a1_, X_b1_, and X_b3_, respectively (**Figures 4E, F, H**).

Concerning group III, the MS^2^ spectrum of m/z 1035 exhibited spiked-signal fragment ions in *Synechococcus* OE as well as in *Synechocystis* WT, but no m/z 748 ion (**Figure 4G**). The m/z 1035 ion in *Synechococcus* OE was thus similar in the MS^2^ spectrum to m/z 1007 lipid X_a1_, but not to m/z 1035 lipid X_b2_, and accordingly was designated as lipid X_a2_. Surprisingly, bubbling-aeration cultured *Synechocystis* cells, distinct from statically-cultured ones, showed m/z 1035 lipid X_a2_ in place of lipid X_b2_, as revealed in its MS^2^ spectrum (**Figure 2E**). One possible explanation might be growth-condition (aeration or static culture)-dependent synthesis of m/z 1035 lipid X_a2_ or lipid X_b2_ in *Synechocystis*, which will, however, require future experimental verification, including elucidation of the chemical structures of both lipid X molecular species. Based on these observations, it was found that *Synechococcus* cells have no intrinsic ability to synthesize lipid X but possess substrates for the synthesis of at least four lipid X molecular species.

**Figure 5:**
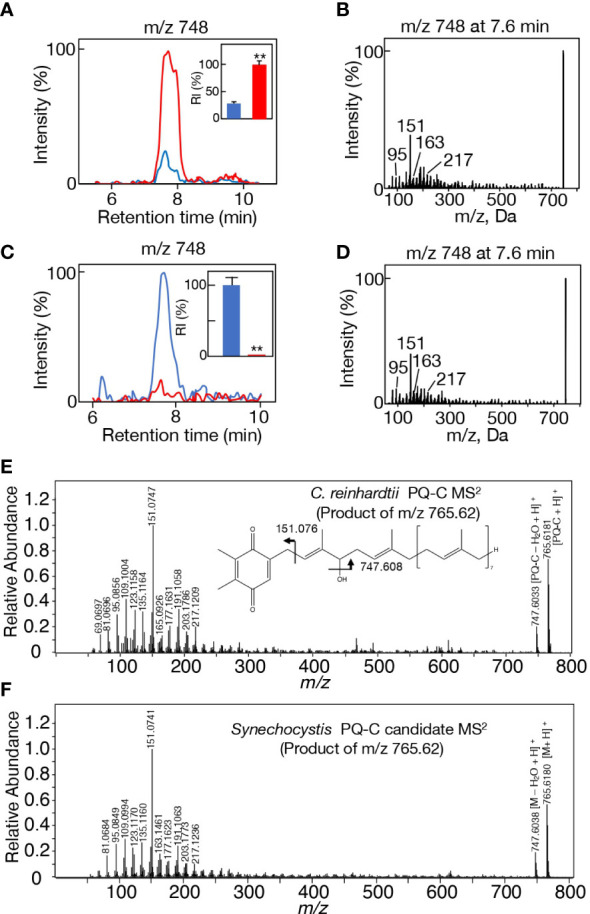
Candidate alcohol that Slr2103 acts in lipid X synthesis. **(A)** MS chromatogram of m/z 748 on LC-MS analysis of total lipids in WT (blue) or Δ*slr2103* (red). The signal intensity of m/z 748 relative to the total lipid fraction in Δ*slr2103* was adjusted to 100%. Inset, the estimated relative signal intensity of m/z 748. **(B)** MS^2^ spectrum of m/z 748 in Δ*slr2103*. **(C)** MS chromatogram of m/z 748 on LC-MS analysis of total lipids in EV (blue) or OE (red) in *Synechococcus*. The signal intensity of m/z 748 relative to the total lipid fraction in EV was adjusted to 100%. Inset, the estimated relative signal intensities of m/z 748. **(D)** MS^2^ spectrum of m/z 748 in EV. **(E)** High-resolution MS^2^ spectrum of H^+^-adducted PQ-C in *C reinhardtii*. The structure of PQ-C with the hydroxy group at the first isoprene unit is tentatively shown. **(F)** High-resolution MS^2^ spectrum of H^+^-adducted Y_765_ in *Synechocystis*. The values shown are averages ± SD for three experiments. The significance of differences was evaluated by means of Student’s *t*-test. **P<0.05.

Simultaneously found on LC-MS analysis was a markedly stronger signal of the m/z 748 ion (here designated as the Y_748_ ion) in Δ*slr2103* than in the WT at 7.4-8.2 min, i.e., much earlier than lipid X (**Figure 5A**). Importantly, the MS^2^ spectrum of Y_748_ at 7.6 min exhibited spiked-signal fragment ions (**Figure 5B**), the top twenty of which in signal intensity coincided with ≧80% of the fragment ions for m/z 1007, 1021, 1035, and 1049, i.e., lipids X_a1_ and X_b1-3_ (**Supplementary Table 1**). It was thus highly probable that Y_748_, which accumulated in Δ*slr2103* was structurally incorporated into lipids X_a_ and X_b_ in the WT, and that the analyte, which Y_748_ was derived from before ion adduction, represents the substrate that Slr2103 utilizes for direct acylation to synthesize lipid X_b_. Consistently with this idea, Y_748_ was detected in the *Synechococcus* EV strain in the LC-MS spectrum of total lipids whereas it became undetectable in its OE strain, owing probably to its exhaustion due to the forced synthesis of lipid X (**Figures 5C, D**).

In this context, it was of note that both m/z 766 and 788 ions were eluted in the same retention time range as that of m/z 748 (7.4-8.2 min) in Δ*slr2103*, with their respective signals as well as the m/z 748 signal being much stronger in Δ*slr2103* than in the WT (**Supplemental Figures 6A, B**). The LC-MS^2^ spectrum of m/z 766 ion showed spiked signals below m/z 300, i.e., characteristic product ions of m/z 748 (**Supplementary Figure 6C**, left). Moreover, the m/z 766 ion showed the m/z 748 ion also as the product ion, which could be explained by that the m/z 748 product ion was generated through a loss of water in the m/z 766 precursor ion. Meanwhile, the m/z 788 ion gave no product ion signals, implying that it represented [M+Na]^+^, as was observed in Na^+^-adducted lipid X (**Supplementary Figure 6C**, right). In view of the same retention time range and higher signal intensities in Δ*slr2103* than in the WT, these three ions, m/z 748, 766, and 788, would be derived from the same analyte that represents the acyl-acceptor substrate of Slr2103. It was thus reasonable to consider that m/z 748, 766, and 788 represent [M-H_2_O+H]^+^, [M+H]^+^, and [M+Na]^+^, respectively, and that the molecular weight of the analyte is 765. The relatively wide retention time range of m/z 748, 766, and 788 ions, together with some apparent peaks in m/z 766 and 788 ions (**Supplementary Figure 6B**), prompted us to investigate the MS^2^ spectrum of the m/z 748 ion at 7.8 or 8.0 min. The MS^2^ spectrum of m/z 748 at 7.8 or 8.0 min was almost the same as that at 7.6 min (**Supplementary Figure 6D**). The analyte, which represents the Slr2103 substrate, would therefore be composed of isomers, and was herein designated as Y_765_.

**Figure 6:**
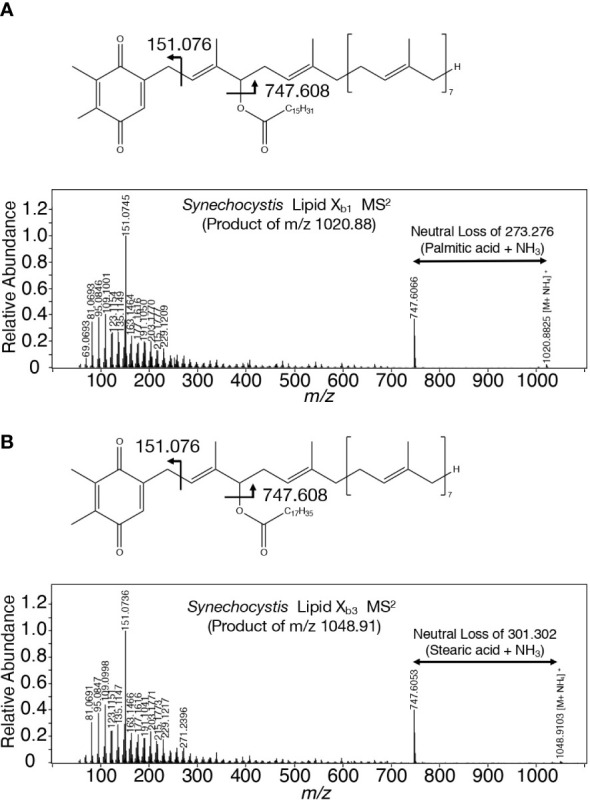
Chemical structures of lipid X_b_ molecules with their high-resolution MS^2^ spectra. **(A)** Palmitoyl PQ-C: lipid X_b1_. **(B)** Stearoyl PQ-C: lipid X_b3_. The structure of acyl PQ-C is tentatively shown to possess the ester bond in the first isoprene unit.

**Figure 7:**
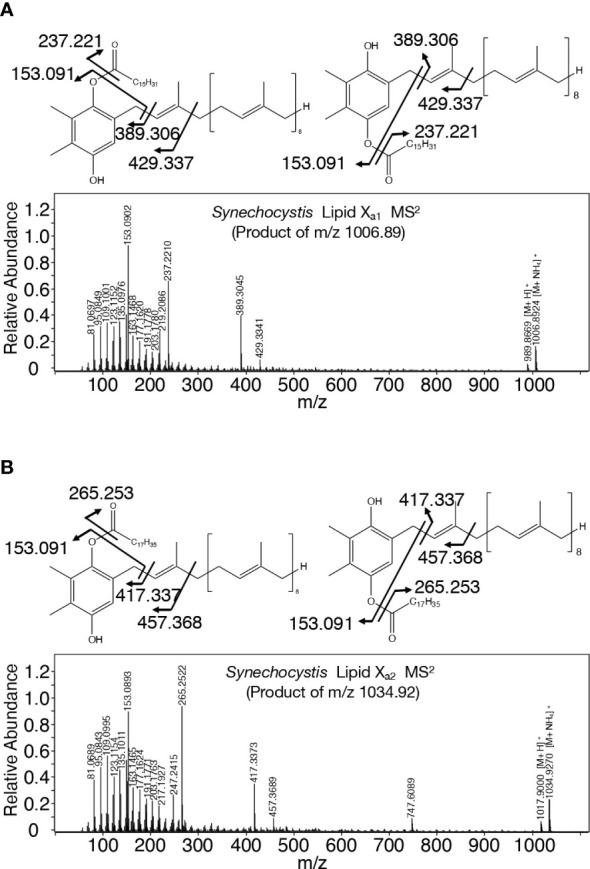
Chemical structure of lipid X_a_ molecules with their high-resolution MS^2^ spectra. **(A)** Palmitoyl plastoquinol: lipid X_a1_. **(B)** Stearoyl plastoquinol: lipid X_a2_.

A search for candidates for isoprenoid-structured alcohols for Y_765_ in photosynthetic organisms or bacteria allowed us to find plastoquinone (PQ)-C (C_53_H_80_O_3_: molecular weight, 765) in seed plants such as *Arabidopsis thaliana* and a green alga, *Chlamydomonas reinhardtii* (Kruk and Szymańska, 2021). No data on the MS^2^ spectrum of PQ-C was available, however, the MS^2^ spectrum of PQ was found to display several common product ions with those of m/z 766 or 748, including m/z 95, 151, 163, and 217 (**Figure 5B, D**, **Supplementary Figure 6C**, **Supplementary Table 1**; Singh et al., 2012). In particular, it was expected that m/z 151 corresponds to a product ion containing a quinone ring (Singh et al., 2012). For more detailed characterization of the chemical structure of Y_765_, we compared its high-resolution MS^2^ spectrum with that of the PQ-C standard. For that purpose, ion-adducted PQ-C was first searched for in *C. reinhardtii* cells (Kruk and Strzałka, 1998; Nowicka and Kruk, 2012; **Supplemental Figures 7A, B**). It was found that PQ-C in *C. reinhardtii* as well as Y_765_ displayed [M-H_2_O+H]^+^, [M+H]^+^, and [M+Na]^+^ ions on high-resolution MS. Moreover, the MS^2^ spectrum of the m/z 765.620 ion, i.e., the H^+^-adducted PQ-C standard, was essentially the same as that of H^+^-adducted Y_765_ (**Figures 5E, F**). These observations therefore demonstrated that Y_765_ is PQ-C, consistent with the presence of isomers in PQ-C as well as in Y_765_, concerning the position of a hydroxy group in the isoprenoid side chain (Kruk and Szymańska, 2021). Collectively, it was deduced that Slr2103 is a PQ-C acyltransferase that esterifies 16:0 or 18:0 with PQ-C for the synthesis of lipid X_b1_ and X_b3_, respectively. Consistent with this idea, the accurate masses of lipids X_b1_ (m/z 1020.887) and X_b3_ (1048.913) were almost the same as the exact masses of the ammonium ([NH_4_]^+^: exact mass, 18.034) adducted palmitoyl PQ-C (C_69_H_110_O_4_: exact mass, 1002.840) and stearoyl PQ-C (C_71_H_114_O_4_: exact mass, 1030.872), respectively (**Figure 6**). The high-resolution MS^2^ spectrum of palmitoyl or stearoyl PQ-C allowed us to confirm product ions that were generated through the neutral loss of the fatty acid and cleavage at the isoprenoid chain with the quinone ring retained (**Figure 6**). In line, the high-resolution MS^2^ spectrum of lipid X_a1_ or X_a2_ was obtained (**Figure 7**).

### Responsibility of *slr2103* for cell growth in a static culture and its enhancement under NaCl-stress conditions

**Figure 8:**
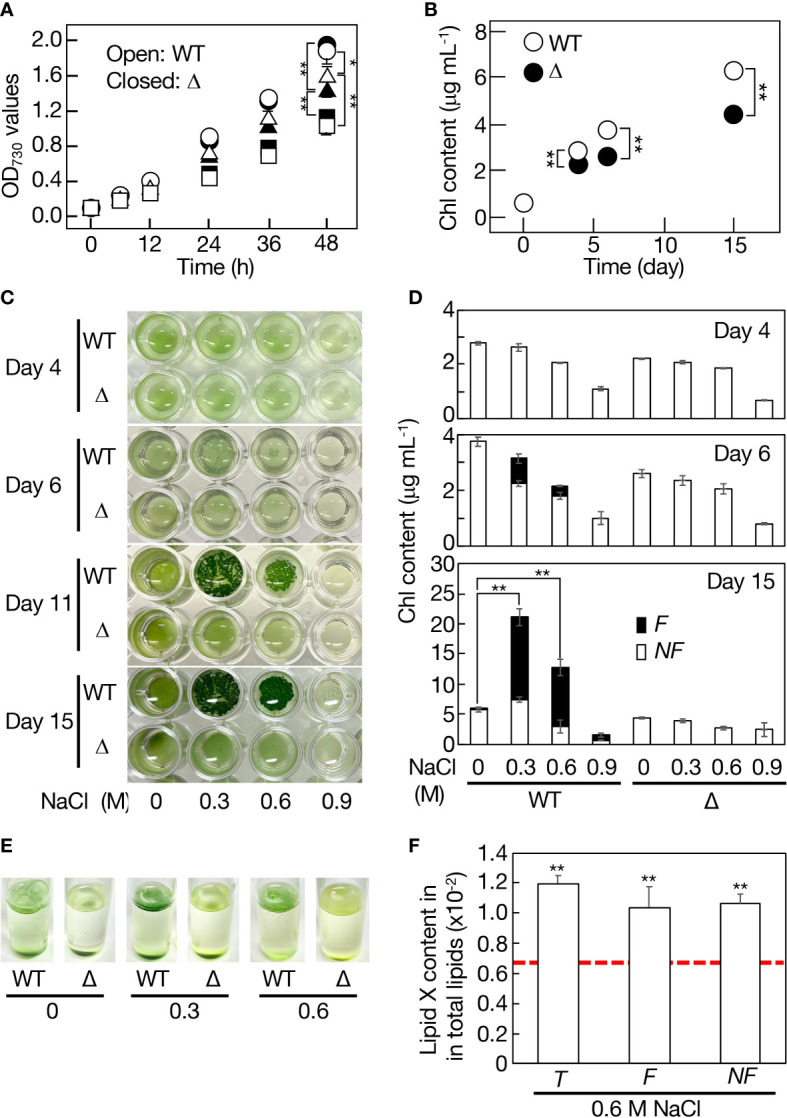
Contribution of *slr2103* to cell growth in *Synechocystis* in a static culture and its enhancement under saline stress conditions. **(A)** Effects of NaCl-stress on cell growth in the WT (open symbols) and Δ*slr2103* (closed symbols) in bubbling aeration cultures. Circles, 0 M NaCl. Triangles, 0.3 M NaCl. Squares, 0.6 M NaCl. **(B)** Cell growth of *Δslr2103* in a static culture. Photographs of microtiter plates where the WT and Δ*slr2103* cells were statically cultured with or without NaCl stress at 0.3 to 0.9 M **(C)**, and concomitant monitoring of cell growth based on the Chl content **(D)**. Open and closed bars correspond to the non-floating (*NF*) and floating (*F*) cells, respectively. **(E)** Photographs of test tubes where the WT and Δ*slr2103* cells were statically cultured for 9 days with 0-0.6 M NaCl stress. **(F)** Quantitative behavior of lipid X relative to total lipids in statically cultured *Synechocystis* cells for 15 days with or without NaCl stress at 0.6 M, which was estimated as in **Figure 1F**. Indicated are the values for total (*T*), *F* or *NF* cells. Red dotted line, initial lipid X content in aeration-cultured cells before the shift to static conditions. The values shown are averages ± SD for three experiments. The significance of differences was evaluated by Student’s *t*-test. *P < 0.1. **P<0.05.

No obvious defect was observed in Δ*slr2103*-cell growth under normal conditions, as previously reported (**Figure 8A**, see control; Aizouq et al., 2020; Tanaka et al., 2020). Meanwhile, Δ*slr2103* was inferior to WT in cell growth when cultured on a non-shaking microtiter plate, i.e., in a static culture (**Figure 8B**). It should be emphasized that this superiority of the WT was due mainly to growth of the sedimented cells (**Figures 8C, D**, see WT at 0 M NaCl).

Cyanobacterial strains that contain *slr2103* homologs, including *Synechocystis*, inhabit freshwater, coastal, or harsh environments like saline or alkaline lakes whereas those with no homologs include oceanic *Synechococcus* and *Prochlorococcus* strains (**Supplemental Table 2**). One of the environmental stresses related with these non-oceanic cyanobacteria but not with oceanic ones might be fluctuating saline stress. *Synechocystis* WT or Δ*slr2103* cells were then subjected to 0.3-0.9 M NaCl stress in aeration-bubbling cultures (**Figure 8A**). Observed in an aeration culture was a NaCl-dose dependent delay in cell growth for the WT or Δ*slr2103*, however, there was no detrimental effect in Δ*slr2103* on an extent of the delay. These results, together with no increase in the lipid X content upon imposition of 0.6 M NaCl stress (**Figure 1G**), indicated no role of lipid X in the cellular acclimatization to NaCl stress in the aeration culture. Meanwhile, in a static culture, addition of NaCl to the WT at 0.3 M, in particular, and 0.6 M also stimulated cell growth such that the total Chl contents, which reflected cell densities, reached 21 and 13 μg Chl mL^-1^, i.e., 3.4- and 2.1-fold higher levels relative to at 0 M NaCl, respectively, on day 15 (**Figures 8C, D**). The growth superiority of the NaCl-stress cells over non-stress ones in a static culture was supported by vigorous proliferation of cells that aggregated to float as a thin layer on the culture surface (**Figures 8C-E**). The bloom-like structure formation began at the latest on day 6 (**Figures 8C, D**). At 0.6 M NaCl, the lipid X content was similar to the initial level irrespective of whether the cells were floating or non-floating, which again ruled out the possibility of lipid X being a storage lipid (**Figure 8F**). In contrast, the NaCl stress had no promoting effect on cell growth or bloom-like structure formation for Δ*slr2103* (**Figures 8C-E**). It thus seemed that in a static culture, lipid X facilitates the growth of sedimented cells (**Figure 8B**), and that the growth-facilitating effect of lipid X becomes pronouncedly greater in NaCl-stress cells through induction of cell aggregation and floatation.

## Discussion

### Taxonomically-biased distribution of lipid X in cyanobacteria

TG is physiologically crucial as an energy- and carbon-storage compound, and is present in eukaryotic cells in general and in prokaryotic cells in certain genera (Alvarez, 2016; Chapman et al., 2019). This study demonstrated that TG is absent in *Synechocystis*, and that instead, it possesses a PQ-related neutral lipid, lipid X, which was found to comprise two subclasses, acyl PQ-C and lipid X_a_, through LC-MS^2^ analysis (**Figures 1**, **2**; **Supplemental Figures 1, 2**). Moreover, it was demonstrated that *slr2103* is the gene for PQ-C acyltransferase, and is essential for the synthesis of a complete set of lipid X molecules, including acyl PQ-C, through both gain-of-function and loss-of-function analyses (**Figure 4**; **Supplemental Figures 1, 5**). During the preparation of this manuscript, a neutral lipid, which showed TG-like TLC mobility, was identified as acyl plastoquinol in Synechocystis on MS and NMR analyses (Mori-Moriyama et al., 2023). Two molecules of the neutral lipid were estimated to be 988.74 and 1016.84 in molecular mass (Mori-Moriyama et al., 2023), and thus correspond to lipids X_a1_ and X_a2_ (989 and 1017, respectively, in nominal mass; **Figures 2A, C**). The high resolution MS^2^ spectra of lipid X_a1_ and X_a2_ supported their identification (**Figure 7**). It might thus be proposed that Slr2103 is a bifunctional acyltransferase that utilizes not only PQ-C but also plastoquinol as an acyl acceptor substrate. For validation of this idea, however, the enzymatic characterization of Slr2103 protein *in vitro* with the use of substrate candidates will be necessary in the future.

Two research groups reported the presence of TG in *Synechocystis* through both TLC and LC-MS^2^ analyses (Aizouq et al., 2020; Tanaka et al., 2020). Tanaka et al. (2020) found that the TG content in *Synechocystis* WT did not greatly surpass the background level, which would be compatible with our finding that TG is absent in this cyanobacterium. Aizouq et al. (2020) performed quantitative analysis of TG relative to OD_750_ in the culture only, it therefore seems necessary to determine the quantitative ratio of TG to PQ lipids that are composed of acyl PQ-C and acyl plastoquinol in their lipid samples, which would be useful for discussion of whether the TG detected represents some contamination or its physiological significance. Notedly, Jimbo and Wada (2023) suggested the rapid turnover of TG in *Synechocystis* through characterization of a disruptant as to the gene for lipase A. Aizouq et al. (2020) proposed that Slr2103 functions in the synthesis of both TG and phytyl esters in *Synechocystis*. Because of the absence of TG in *Synechococcus* OE as well as in *Synechocystis* WT (**Figures 1A**, **4C**), it was shown that *slr2103* cannot contribute to TG synthesis under our experimental conditions. Concerning phytyl esters, the role of *slr2103* in their synthesis was proposed based on observations including only a partial reduction in its content in Δ*slr2103* relative to in the WT. It can thus be deduced that Slr2103 functions mainly in PQ-lipid synthesis. Distinct from cyanobacteria, plants have long been known to contain PQ-C and/or acyl PQ-C, i.e., PQ-B (Kruk and Szymańska, 2021). PQ-C is produced non-enzymatically from PQ through the direct action of singlet oxygen (^1^O_2_), one of the reactive oxygen species. Concerning the plant PQ-B, its acyl-moiety was reported to comprise mainly saturated fatty acids like 16:0, similar to *Synechocystis* counterparts (Kruk et al., 1998). Our identification of the gene for cyanobacterial PQ-C acyltransferase would provide a clue to elucidate the PQ-B synthetic process in plants, including the plant-type PQ-B synthesis genes.

Slr2103, as compared with authentic DGAT2, showed modified motifs and the lack of a membrane spanning region (**Supplemental Figure 3**). In line, Slr2103 belonged to a cyanobacterial clade that is far from eukaryotic clades of authentic DGAT2, including those of plants and algae, in the molecular phylogenetic tree (**Figure 3**). These peculiar structural properties of Slr2103 would explain its utilization of PQ-C but not DG. Of further note is the taxonomically-biased distribution of *slr2103* homologs in cyanobacteria, which is represented by exclusion of *slr2103* homologs in oceanic *Prochlorococcus* and *Synechococcus* species (**Table S2**). Some ancestor of *slr2103* homologs might have been acquired by ancient cyanobacteria, and thereafter inherited by only some cyanobacteria through their evolutionary diversification, with functional development for the synthesis of PQ lipids.

In view of the lack of orthologs of known TG-synthesis genes such as *DGAT* series in cyanobacteria and no contribution of *slr2103* to the synthesis of TG in cyanobacteria (**Figures 1A**, **4C**), it would be reasonable to consider that cyanobacteria do not synthesize TG; instead, only cyanobacterial stains having *slr2103* homologs synthesize PQ lipids as in *Synechocystis*: i.e., it is probable that in cyanobacterial strains, the genome base sequences of which have been reported, 103 strains with *slr2103* homologs possess PQ lipids whereas the other 265 strains without such a homolog, including oceanic *Prochlorococcus* and *Synechococcus* strains, do not have it. For evaluation of this idea, it will be necessary to investigate the chemical structure of neutral lipids and/or the functions of *slr2103* homologs through gene manipulation in homolog-containing cyanobacteria.

### Essentiality of PQ lipids for formation of biofilms or that of NaCl-stress induced bloom-like structure in a static culture

Cyanobacteria inhabit a wide range of environments, including deserts and the cryosphere, and, particularly, some selected strains, owing to their established genetic tools, have thus far been subjected to numerous studies as to environmental-stress physiology (Rachedi et al., 2020). One subject of such study series is the mechanism of acclimatization to saline stress in *Synechocystis* with useful genetic tools, including gene disruption and introduction systems involving natural transformation: the mechanism has been shown to include modulation of gene expression for compatible-solute synthesis, ionic homeostasis, and regulatory asRNAs and sRNAs (Hagemann, 2011; Klähn et al., 2021). However, these studies utilized *Synechocystis* cells in aeration cultures with agitation or air-bubbling, which would not have allowed researchers to observe the ability of the cells to form a bloom-like structure or its physiological merit, as in our experiments shown in **Figure 8A**. Meanwhile, there was a report of NaCl-stress induced bloom formation in statically-cultured cells of *Microcystis aeruginosa*, which, however, provided no insight into its physiological significance or molecular mechanism (Dervaux et al., 2015). The NaCl-stress induced expansion of the bloom-like structure in *Synechocystis* would be achieved through better photosynthesis performance with an efficient supply of atmospheric CO _2_ to cells on the culture surface, relative to non-NaCl stressed sedimented cells. The formation of the bloom-like structure in a static culture of *Synechocystis*, including the essential role of *slr2103* in the synthesis of PQ lipids, can thus be regarded as a novel strategy to promote NaCl-stress acclimatizing cell growth in cyanobacteria. Future study will include elucidation of how PQ lipids or *slr2103* contributes to the acclimatization, including whether it acts directly or indirectly. Although PQ-B has long been recognized in plants as a minor lipid component, information on its physiological roles is limited, including the possible electron acceptor from PSII and its quantitative increase with age in *A. thaliana* when grown under high light conditions (Kruk et al., 1998; Dłużewska et al., 2015). The indispensable roles of PQ lipids including PQ-B in cyanobacterial NaCl-acclimatization would facilitate reexamination of the physiological roles of PQ-B in plants.

In non-tuberculous *Mycobacterium smegmatis* cells, a species-specific neutral lipid, monomeromycolyl diacylglycerol, which is present at the cell surface, is supposed to facilitate cell flocculation through a hydrophobic interaction for the formation of a pellicle biofilm or bloom-like structure (Chakraborty and Kumar, 2019). Interestingly, *M. smegmatis* possesses a DGAT2 homolog that was included in a bacterial clade that is close to the cyanobacterial one in the molecular phylogenetic tree (**Figure 3**). Future goals will be elucidation of the molecular mechanism by which PQ lipids help form sedimented-cell communities in static *Synechocystis* culture, and the bloom-like structure for acclimatization to NaCl stress, including the possibility of PQ lipids participating in the construction of the cell-surface network. In this context, it was of particular interest that statically-cultured cells, as compared with aeration-cultured ones, accumulated lipid X much more abundantly (**Figure 1H**).

*Synechocystis* is also a promising cyanobacterial bioresource for biofuels and biodegradable plastics (Agarwal et al., 2022). The harvesting process for photosynthetic microbes for high-value added products includes biomass concentration, the system cost of which is now too high due to the requirement of large amounts of chemicals or excessive electric power and expensive equipment for cell floatation with air-bubbling (Min et al., 2022). It is thus necessary to economize the biomass-concentrating systems of photosynthetic microbes. The elucidation of the molecular mechanism by which PQ lipids help form biofilms or the NaCl-induced bloom-like structure, as described above, would lead to enhanced NaCl-induced cell aggregation and/or floatation for *Synechocystis*, including shortening of the time necessary for expansion of the bloom and enhancement of the population ratio of floating to non-floating cells, through gene manipulation. A new avenue will thereby be opened to develop economical biomass concentrating systems that utilize abundant seawater at a low cost, not only for *Synechocystis* but also for algal or other cyanobacterial species of high industrial value through co-culture flocculation/floatation with *Synechocystis* cells (Min et al., 2022). Meanwhile, as has been typically reported for *Microcystis* species that excrete microcystins toxic to the liver, the bloom water harms our health when utilized for human activities such as drinking (Harke et al., 2016). It is generally accepted that *Microcystis* cells, distinct from *Synechocystis* ones, utilize intracellular proteinaceous gas vesicles filled with air for cell floatation in blooming (Pfeifer, 2022). It is of note that cyanobacterial species that naturally bloom like *Microcystis* contain *slr2103* homologs (**Table S2**). Study of the molecular mechanism of PQ-lipid function in the bloom-like structure formation of *Synechocystis*, based on our novel information, would provide clues for a better understanding of the molecular mechanism of natural blooming in *Microcystis* and accordingly clues to control this cyanobacterial blooming.

## Data availability statement

The raw data supporting the conclusions of this article will be made available by the authors, without undue reservation.

## Author contributions

MK: Investigation, Methodology, Validation, Writing – review & editing. MA: Investigation, Methodology, Validation, Writing – review & editing. KH: Investigation, Methodology, Validation, Writing – review & editing. TS: Investigation, Validation. RI: Investigation. MT: Supervision. NS: Conceptualization, Writing – original draft and – review & editing, Supervision. All authors contributed to the article and approved the submitted version.
