# Supplementary material for: slr2103, a homolog of type-2 diacylglycerol acyltransferase genes, for plastoquinone-related neutral lipid synthesis and NaCl-stress acclimatization in a cyanobacterium, Synechocystis sp. PCC 6803
Source: Front Plant Sci. 2023 Apr 26;14:1181180. doi: 10.3389/fpls.2023.1181180 (PMC10171310; doi:10.3389/fpls.2023.1181180)
Supplement: Supplementary file 3 [file DataSheet_1.pdf]

Supplemental Figures:

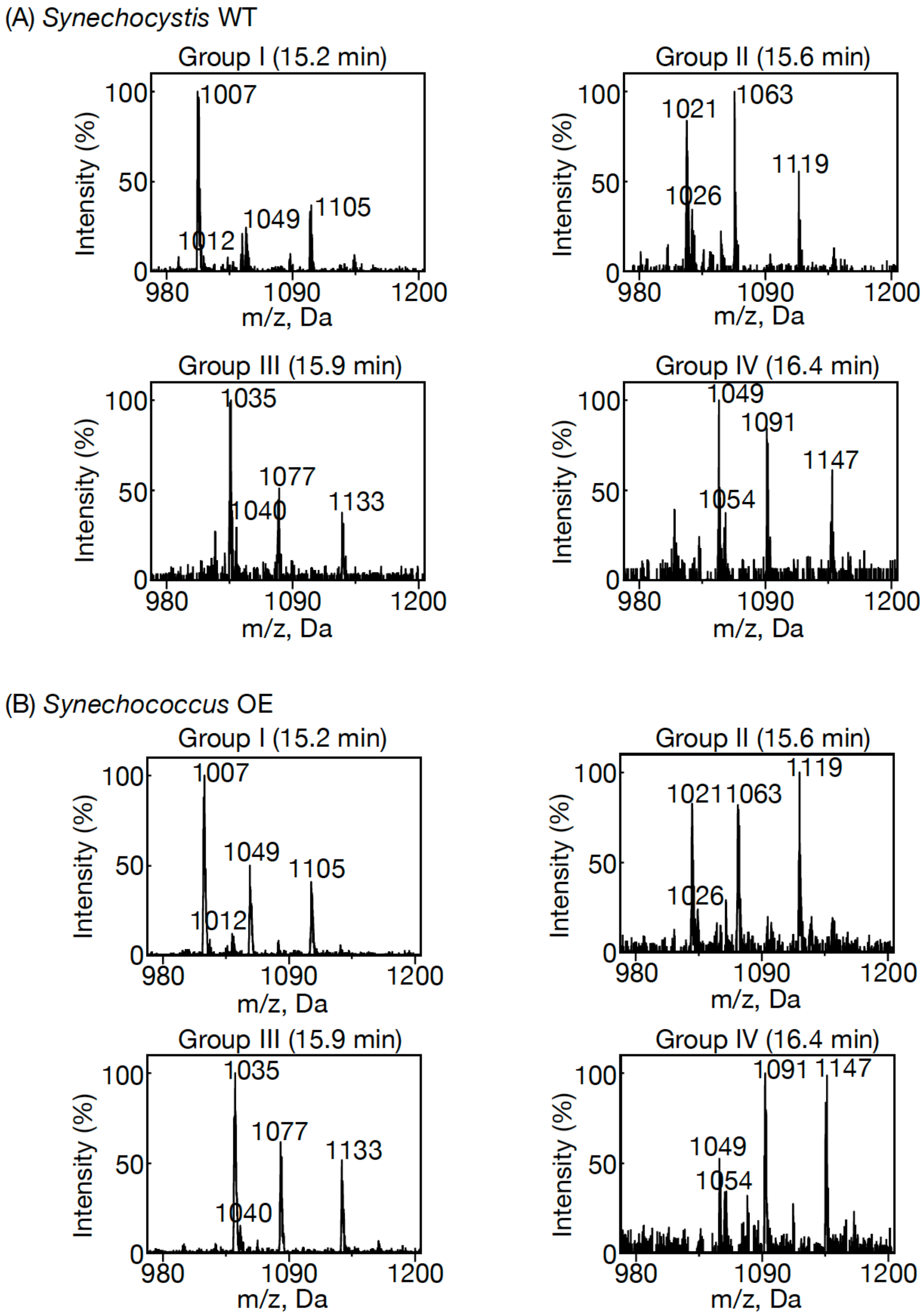

Figure S1. Classification of lipid X molecules into four groups, I-IV, as to retention time on LC-MS analysis. Total cellular lipids were analyzed in *Synechocystis* WT in a static culture (A) and in *Synechococcus* OE in an aerated culture (B).

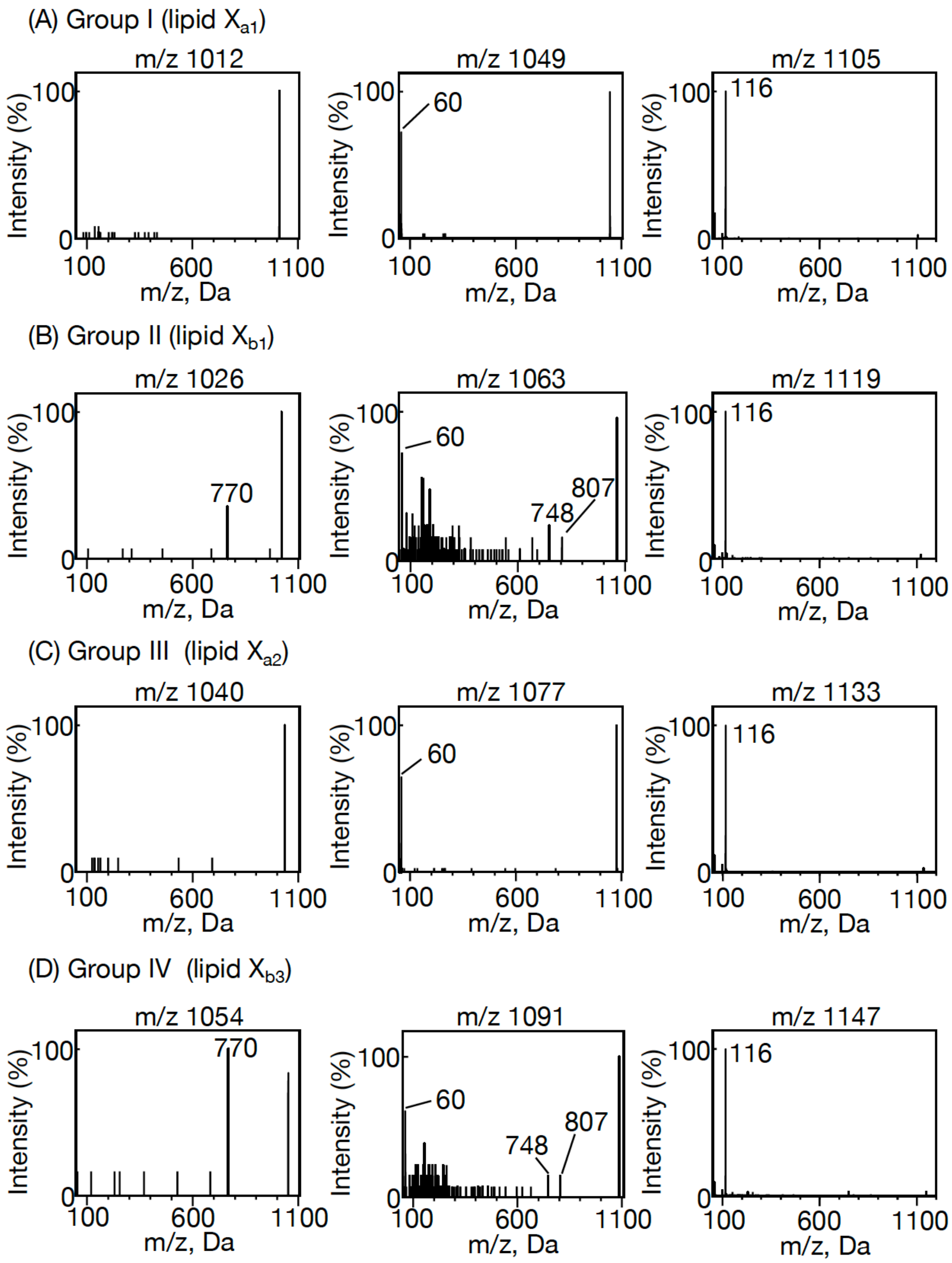

Figure S2. MS^2 spectra of individual lipid X molecules in statically-cultured *Synechocystis* cells. Three members of group I (A), II (B), III (C), or IV (D) in *Synechocystis* were subjected to LC-MS^2 analysis.

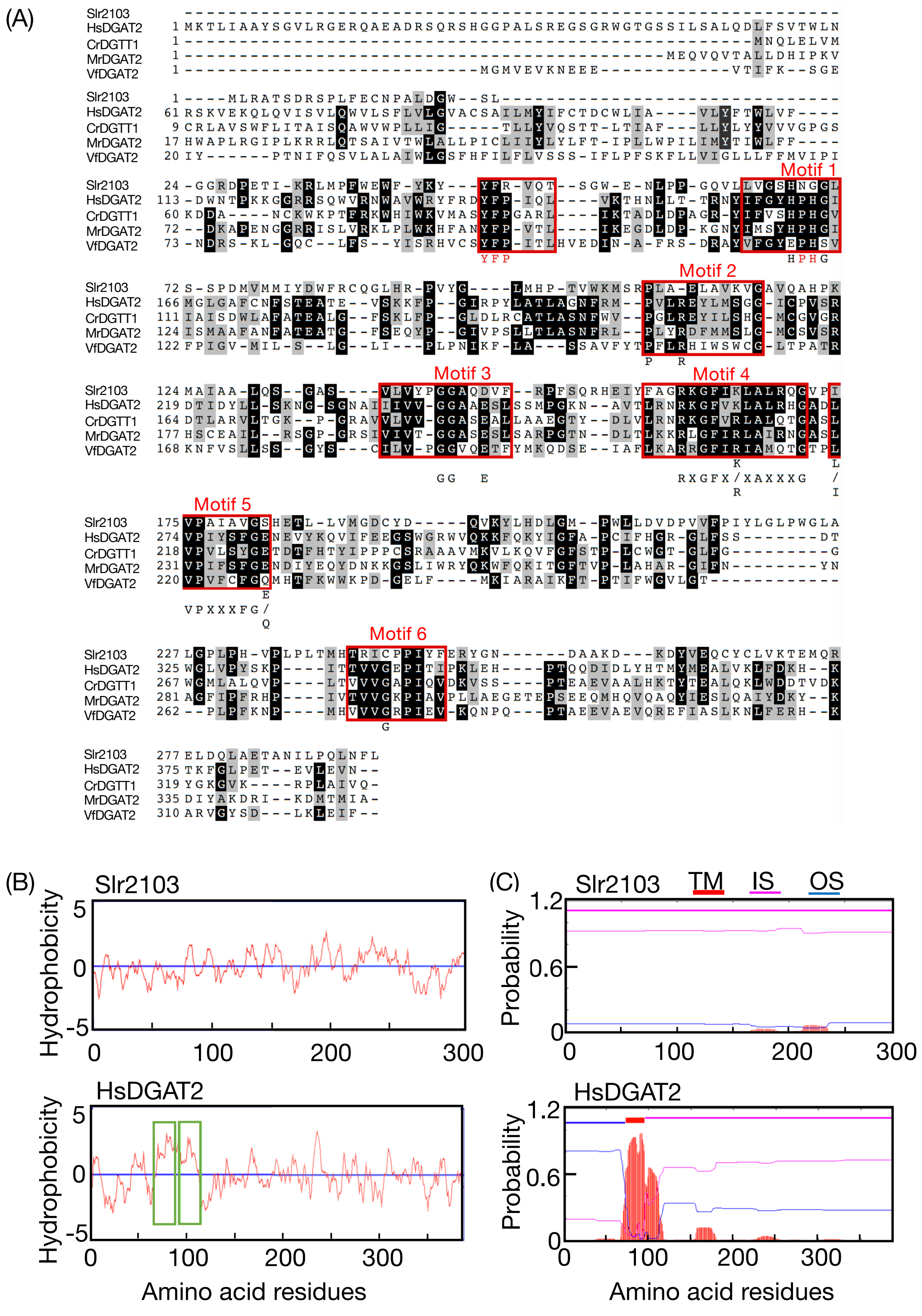

Figure S3. Characterization of the amino acid sequence of the Slr2103 protein. (A) Amino acid sequence alignment of Slr2103 with DGAT2 homologs: HsDGAT2 from

human (Cases et al., 2001), CrDGTT1 from a green alga, *C. reinhardtii* (Hung et al., 2013), MrDGAT2 from a fungus, *Mortierella ramanniana* (Lardizabal et al., 2001), and VfDGAT2 from a seed plant, *Vernicia fordii* (Shockey et al., 2006). (B) Comparison of the hydrophobicity of Slr2103 with that of HsDGAT2, according to SOSUI (Imai et al., 2008). (C) Predicted transmembrane helices not in Slr2103 but in HsDGAT2, as seen on TMHMM analysis (Krogh et al., 2001). TM, transmembrane. IS, inside. OS, outside.

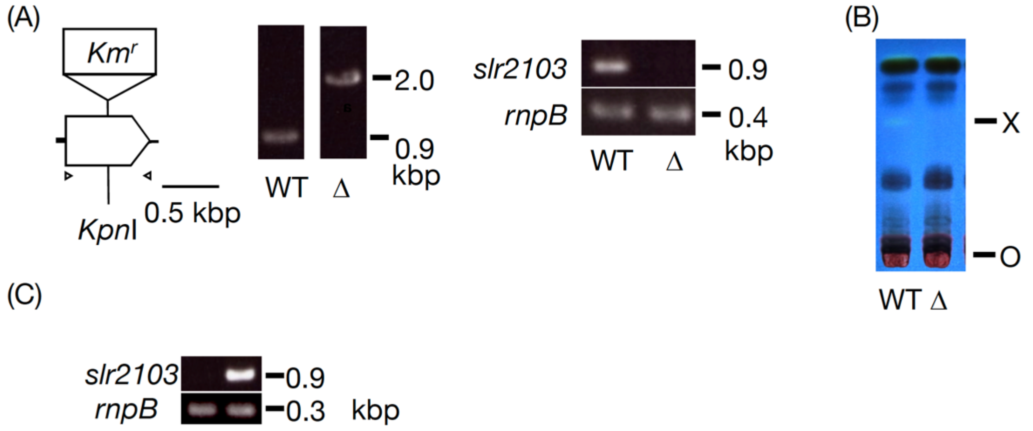

Figure S4. Manipulation of *slr2103* expression in *Synechocystis* or *Synechococcus*, related to Figure 4 and Supplemental experimental procedures. (A) Insertional mutagenesis strategy (left) and confirmation of *slr2103* disruption by PCR with genomic DNA as a template (center) or by semiquantitative PCR with isolated RNA as a template (right) in *Synechocystis*. The *rnpB* gene was used as an internal control in the semiquantitative PCR analysis. (B) The TLC profiles of total lipids in *Synechocystis* WT and Δ*slr2103* with a solvent system of hexane/diethyl ether/acetate (70:30:1, by vol.). X, lipid X. O, origin. (C) Confirmation of *Synechococcus* OE generation by semiquantitative PCR with isolated RNA as a template. As an internal control, *rnpB* was used in the analysis. EV, a control strain that was transformed with the empty vector.

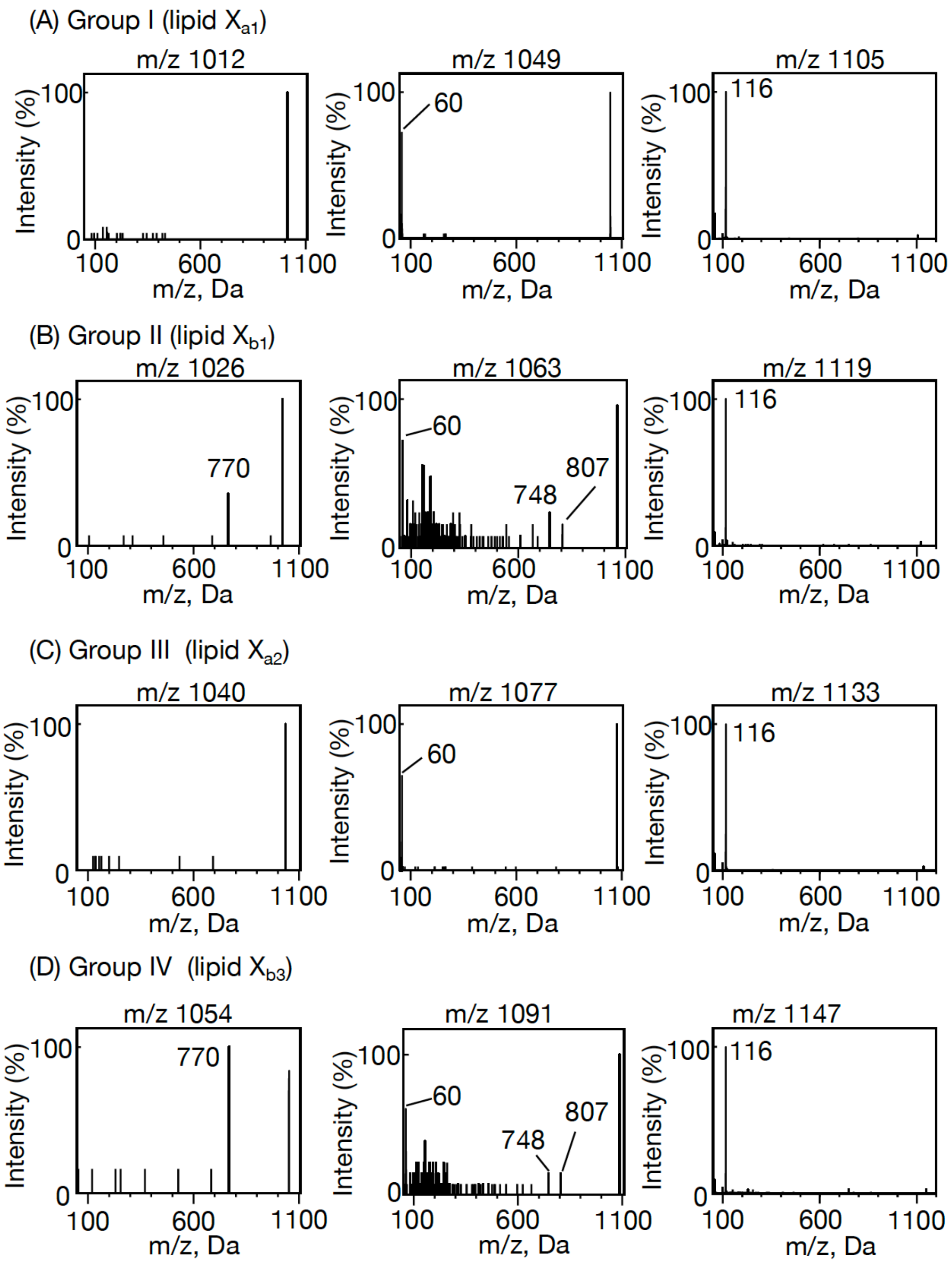

Figure S5. MS^2 spectra of individual lipid X molecules in aeration-cultured *Synechocossus* OE cells, related to Figure 4. Three members of group I (A), II (B), III (C), or IV (D) in *Synechococcus* OE were subjected to LC-MS^2 analysis.

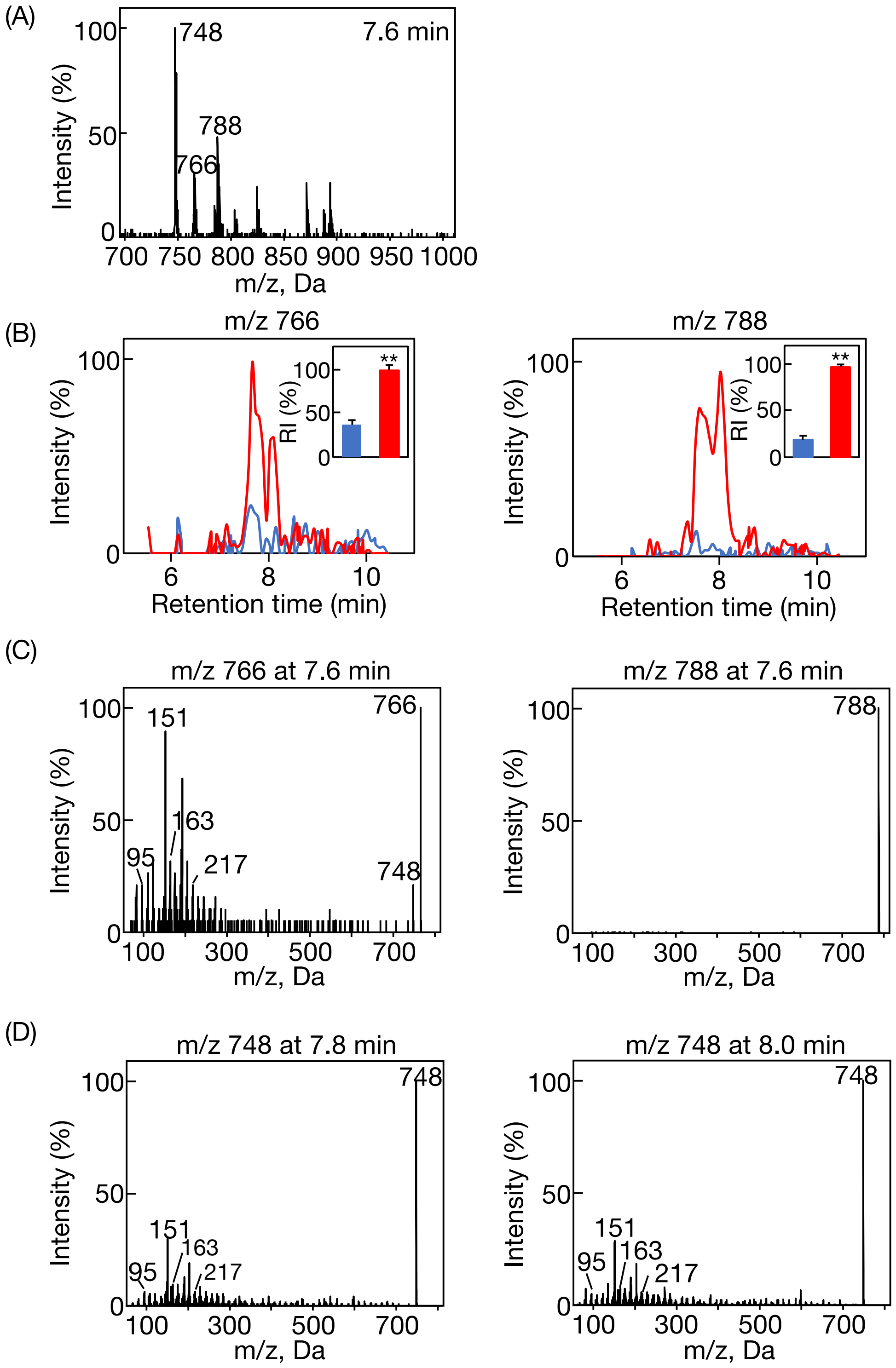

Figure S6. Effects of Δ*slr2103* on the content of an acyl-acceptor substrate candidate of Slr2103. (A) A prominent signal at m/z 748 concomitant with two weaker signals at

m/z 766 and 788 at 7.6 min on LC-MS analysis of total lipids in Δ*slr2103*. Much stronger signal intensity in Δ*slr2103* (red) than in the WT (blue) at m/z 766 (B, left) or m/z 788 (B, right) on LC-MS analysis of total lipids. The MS^2 spectrum of m/z 766 (C, left) or m/z 788 (C, right) in Δ*slr2103*, which was eluted at 7.6 min on LC-MS analysis. The MS^2 spectrum of m/z 748 in Δ*slr2103*, which was eluted at 7.8 (D, left) or 8.0 min (D, right) on LC-MS analysis. The values shown are averages ± SD for three experiments. The significance of differences was evaluated by means of Student's *t*-test. **$P<0.05$.

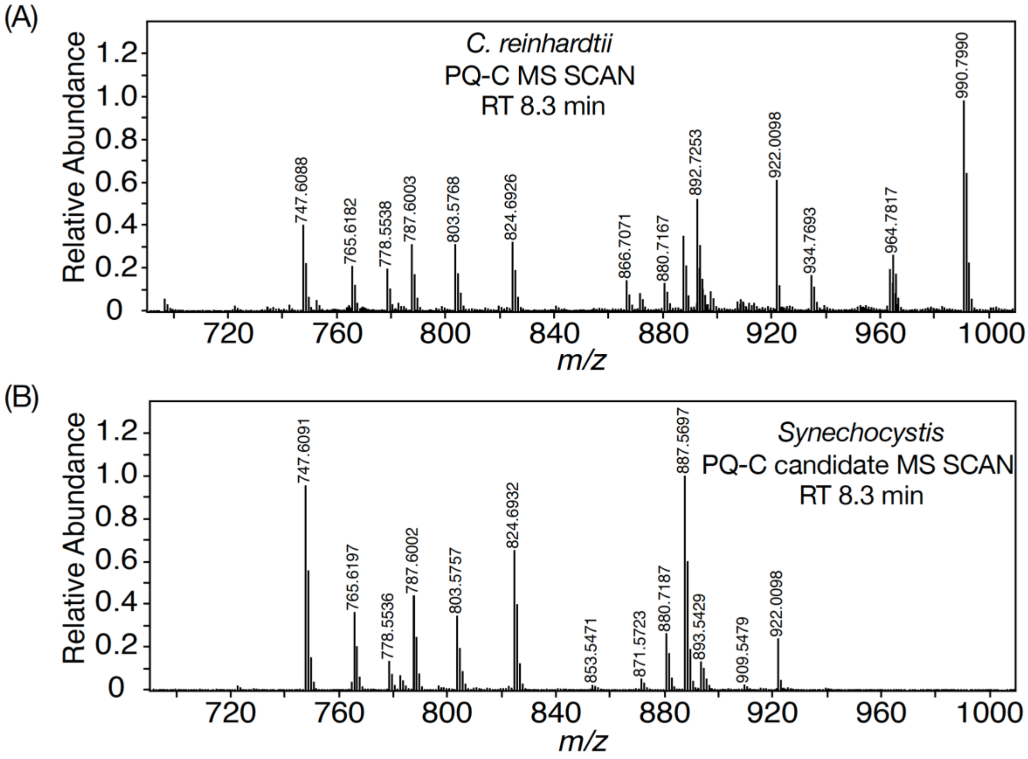

Figure S7. High-resolution LC-MS spectrum of PQ-C or Y_{765}. Ion-adducted PQ-C was searched for in *C. reinhardtiii* cells where the PQ-C content was expected to increase with illumination with strong light (Nowicka and Kruk, 2012), based on both the elution pattern of PQ-C relative to those of Chl *a* and β-carotene (Kruk and Strzałka, 1998) and its exact mass of 764.6107, on high-resolution LC-MS analysis. For that purpose, *C. reinhardtii* cells were aeration-cultured in 3/10 HSM (Sato et al., 2014) in the same manner as in cyanobacteria in Materials and methods. The cells grown to the logarithmic phase were then exposed to strong light (2,000 μmol photons m^{-2} s^{-1}, metal halide lamp) for 2 h. Found were three types of ion-adducted PQ-C $[M-H_2O+H]^+$, $[M+H]^+$, and $[M+Na]^+$ (A), which completely matched the results for Y_{765} (B). Signals with m/z-values represent those at relatively strong intensities.

Supplemental Tables:

Table S1. Coverage of fragment ions of Y_{748} by lipid X in *Synechocystis* or *Synechococcus* OE on product ion analysis, related to Figures 2, 4, and 5.

	M_{748a}	m/z 1007		m/z 1021		m/z 1035		m/z 1049	
		6803	7942	6803	7942	6803	7942	6803	7942
1	m/z 151.2	—	—	+	+	+	—	+	+
2	m/z 191.3	+	+	+	+	+	+	+	+
3	m/z 203.3	+	+	+	+	+	+	+	+
4	m/z 189.2	—	—	+	+	+	—	+	+
5	m/z 135.1	+	+	+	+	+	+	+	+
6	m/z 187.3	+	+	+	+	+	+	+	+
7	m/z 149.2	+	+	—	+	+	+	+	+
8	m/z 217.4	+	+	+	+	+	+	+	+
9	m/z 255.4	+	+	+	+	+	+	+	+
10	m/z 81	+	+	+	+	+	+	+	+
11	m/z 109.1	+	+	+	+	+	+	+	+
12	m/z 147.4	+	+	—	+	+	+	+	+
13	m/z 229.4	+	+	+	+	+	+	+	+
14	m/z 95.0	+	+	+	+	+	+	+	+
15	m/z 163.3	—	+	+	+	+	+	+	+
16	m/z 175.2	+	+	+	+	+	+	+	+
17	m/z 177.4	+	+	+	+	+	+	+	+
18	m/z 203.0	—	—	+	—	—	—	—	—
19	m/z 205.2	+	+	—	+	+	+	+	+
20	m/z 231.4	+	+	+	+	+	+	+	+
Coverage (%)		80	85	85	95	95	85	95	95

Shown is the presence (+) or absence (-) of the top twenty fragment ions of Y_{748} at high signal intensity in the product ion spectrum of lipid X in *Synechocystis* WT (6803) or *Synechococcus* OE (7942).
